# Bis[*cis*-bis­(diphenyl­phosphino)ethene-*κ* ^2^ *P*,*P*′]copper(I) tetra­fluoridoborate ethanol solvate

**DOI:** 10.1107/S1600536809012707

**Published:** 2009-04-08

**Authors:** Peter C. Healy, Bradley T. Loughrey, Michael L. Williams

**Affiliations:** a Eskitis Institute for Cell and Molecular Therapies, Griffith University, Brisbane 4111, Australia

## Abstract

In the title compound [Cu(C_26_H_22_P_2_)_2_]BF_4_·C_2_H_5_OH, a disordered ethanol solvate molecule and the anions are located in well defined channels along the *c* axis. The four-coordinate Cu(P—P)_2_ core of the cation adopts approximately *D*_2_ point group symmetry with the Cu—P bond lengths spanning a narrow range from 2.272 (1) to 2.285 (1) Å.

## Related literature

For the cytotoxic and anti­tumor activity of adducts of the bidentate phosphine ligand Ph_2_P(CH=CH)PPh_2_ with copper, silver and gold(I) salts, see: Berners-Price *et al.* (1985 ▶, 1987 ▶, 1990 ▶). For the structurally related copper(I) and gold(I) PF_6_ complexes, see: Berners-Price *et al.* (1992 ▶). For the angular distortion of the *M*(*L*—*L*)_2_ core of four-coordinate bis­(bidentate) complexes, see: Dobson *et al.* (1984 ▶); Healy *et al.* (2008 ▶).
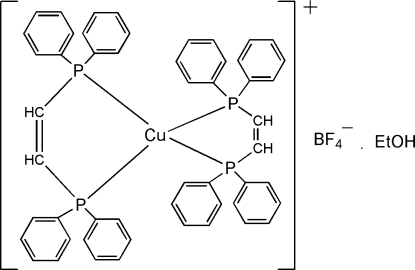


## Experimental

### 

#### Crystal data

[Cu(C_26_H_22_P_2_)_2_]BF_4_·C_2_H_6_O
*M* *_r_* = 989.18
Orthorhombic, 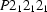

*a* = 14.147 (2) Å
*b* = 16.5719 (11) Å
*c* = 20.9536 (13) Å
*V* = 4912.4 (8) Å^3^
*Z* = 4
Mo *K*α radiation
μ = 0.63 mm^−1^
*T* = 223 K
0.32 × 0.32 × 0.25 mm

#### Data collection

Oxford Diffraction GEMINI S Ultra diffractometer
Absorption correction: multi-scan (*CrysAlis RED*; Oxford Diffraction, 2007 ▶) *T*_min_ = 0.824, *T*_max_ = 0.859
18204 measured reflections
8649 independent reflections
7257 reflections with *I* > 2σ(*I*)
*R*_int_ = 0.031

#### Refinement

*R*[*F* ^2^ > 2σ(*F* ^2^)] = 0.041
*wR*(*F* ^2^) = 0.108
*S* = 0.99
8649 reflections
580 parameters
H-atom parameters constrained
Δρ_max_ = 0.60 e Å^−3^
Δρ_min_ = −0.32 e Å^−3^
Absolute structure: Flack (1983 ▶), 3798 Friedel pairs
Flack parameter: −0.003 (12)

### 

Data collection: *CrysAlis CCD* (Oxford Diffraction, 2007 ▶); cell refinement: *CrysAlis RED* (Oxford Diffraction, 2007 ▶); data reduction: *CrysAlis RED*; program(s) used to solve structure: *SIR97* (Altomare *et al.*, 1999 ▶); program(s) used to refine structure: *SHELXL97* (Sheldrick, 2008 ▶); molecular graphics: *ORTEP-3 for Windows* (Farrugia, 1997 ▶); software used to prepare material for publication: *PLATON* (Spek, 2009 ▶).

## Supplementary Material

Crystal structure: contains datablocks global, I. DOI: 10.1107/S1600536809012707/ng2567sup1.cif

Structure factors: contains datablocks I. DOI: 10.1107/S1600536809012707/ng2567Isup2.hkl

Additional supplementary materials:  crystallographic information; 3D view; checkCIF report

## Figures and Tables

**Table:** 

Cu—P1	2.2775 (10)
Cu—P2	2.2724 (11)
Cu—P3	2.2820 (10)
Cu—P4	2.2851 (10)

**Table:** 

P1—Cu—P2	89.76 (4)
P1—Cu—P3	110.05 (3)
P1—Cu—P4	130.20 (3)
P2—Cu—P3	131.21 (3)
P2—Cu—P4	110.94 (3)
P3—Cu—P4	89.97 (3)

**Table 2:** Hydrogen-bond geometry (Å, °)

*D*—H⋯*A*	*D*—H	H⋯*A*	*D*⋯*A*	*D*—H⋯*A*
O1—H1⋯F4	0.8800	2.1200	2.998 (9)	179.00

**Fig. 1.**
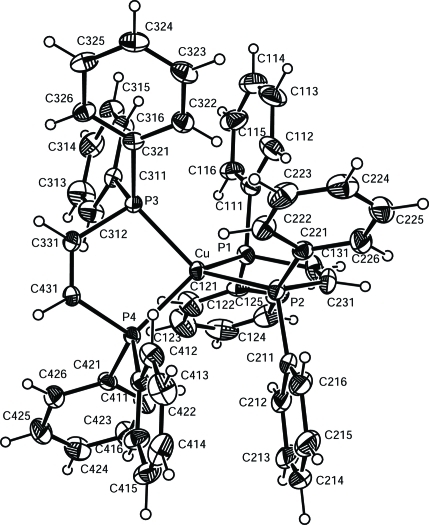
View of the cation of the title complex with the atom numbering scheme. Displacement ellipsoids for non-H atoms are drawn at the 30% probability level.

**Fig. 2.**
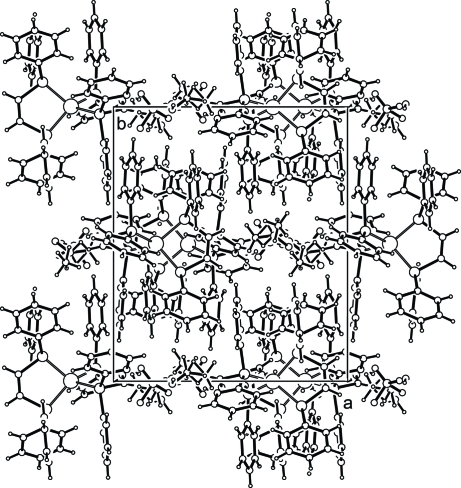
Lattice structure of the title compound viewed down the crystallographic c axis.

## supplementary crystallographic information

### Comment

Adducts of the bidentate phosphine ligand, Ph_2_P(CH=CH)PPh_2_ (dppey), with copper, silver and gold(I) salts form stable bis-chelated ionic complexes [*M*(dppey)_2_]*X* which have been shown to exhibit significant cytotoxic and antitumor activity (Berners-Price *et al.*, 1987; 1985; 1990). In this present study on the synthesis and characterization of copper(I) complexes with this ligand, we have found that addition of aqueous HBF_4_ to a suspension of dppey and a slight stoichiometric excess of copper(I) oxide in ethanol results in precipitation of well formed crystals which were shown by ^1^H NMR, ES Mass Spectrometry and single-crystal X-ray structural analysis to be [Cu(dppey)_2_]BF_4_.EtOH. The crystal structure of this complex was found to be isomorphous with the copper(I) and gold(I) PF_6_ complexes (Berners-Price *et al.*, 1992). A view of the of the [Cu(dppey)_2_]^+^ cation is shown in Fig. 1, with crystal packing viewed down the *c* axis shown in Fig. 2.

In this lattice, the anions and disordered solvent occupy well defined channels along the crystallographic *c* axis, with extensive C—H···F weak bonding interactions between the phenyl and ethene H atoms and the anion fluorides. For the present structure, an O—H···F hydrogen bonding interaction exists between the ethanol hydroxyl proton and the BF_4_ anion.

In the Cu(P—P)_2_ core of the cation, the Cu—P bond lengths span a narrow range from 2.272 (1) - 2.285 (1)Å and are similar to values of 2.276 (2) - 2.289 (2)Å reported for the PF_6_ complex (Berners-Price *et al.*, 1992). The P—Cu—P inter-ligand angles range from 110.08 (4)° to 131.20 (3)° with the P—Cu—P intra-ligand 'bite' angles 89.76 (4) and 89.95 (4)°. In this cation, each ligand adopts a sterically comfortable conformational structure with the phenyl ring planes in each ligand approximately orthogonal to each other, maximizing C—H..π interactions. Charge transfer π···π interactions between phenyl rings of each ligand are likely to contribute to the overall stability of both the cation and the lattice structure.

Angular distortion of the *M*(L—*L*)_2_ core of four-coordinate bis(bidentate) complexes can be conveniently described by the parameters θ*_x_*, θ*_y_* and θ*_z_* which define the orientation of the L—M—*L* plane of ligand 1 with respect to that of ligand 2 (Dobson *et al.*, 1984; Healy *et al.*, 2008). θ*_x_* and θ*_y_* represent rocking motions of the two ligands with respect to each other while θ*_z_* is a measure of the degree of twist between the two planes. For moieties adopting D_2 d_ point group symmetry θ*_x_* = θ*_y_* = θ*_z_* = 90°. In this present structure, θ*_x_* = 90.5°, θ*_y_* = 89.7° and θ*_z_* = 72.7° with the deviation θ*_z_* from 90°, representing a lowering of the point group symmetry of the core from D_2 d_ to approximately D_2_.

### Experimental

1 ml of concentrated aqueous HBF_4_ was added to a suspension of Cu_2_O (0.02 g, 0.14 mmol) and dppey (0.20 g, 0.50 mmol) in 5 ml e thanol. The resultant mixture was heated with stirring to yield a clear pale green solution. Filtration and cooling to room temperature resulted in formation of a crystalline precipitate. Recrystallization by slow evaporation of an ethanol solution of the complex resulted in well formed crystals suitable for X-ray diffraction studies. NMR: ^1^H (d_6_DMSO), δ 1.05 t, J = 6.8 Hz, 3H, CH_3_), 3.43 (dq, J = 7.2 Hz, 7.2 Hz 2H, CH_2_), 7.11–7.14 (m, 16H, C_6_H_5_*ortho*), 7.16–7.20 (m, 16H, C_6_H_5_*meta*), 7.37–7.41 (m, 8H, C_6_H_5_*para*), 7.96–8.07(m, 4H, CH). ESMS (m/*z*): +ve ion, calcd m/*z* for [Cu(dppey)_2_]^+^ 856.46, found 854.95 (100%), -ve ion, calcd m/*z* for BF_4_]^-^ 86.82, found 86.51 (100%). *M*.p. 176–177°C.

### Refinement

H atoms attached to carbons were constrained as riding atoms, with C–H set to 0.94–96 Å. The hydroxyl proton placed at a calculated position along O···F axis and constrained as a riding atom with O–H set to 0.88 Å. *U*_iso_(H) values were set to 1.2*U*_eq_ of the parent atom. The carbon atoms disordered ethanol molecule were refined isotropically.

### Crystal data

**Table:**

[Cu(C_26_H_22_P_2_)_2_]BF_4_·C_2_H_6_O	*F*(000) = 2048
*M**_r_* = 989.18	*D*_x_ = 1.329 Mg m^−^^3^
Orthorhombic, *P*2_1_2_1_2_1_	Mo *K*α radiation, λ = 0.71070 Å
Hall symbol: P 2ac 2ab	Cell parameters from 8876 reflections
*a* = 14.147 (2) Å	θ = 2.9–25.0°
*b* = 16.5719 (11) Å	µ = 0.63 mm^−^^1^
*c* = 20.9536 (13) Å	*T* = 223 K
*V* = 4912.4 (8) Å^3^	Prism, colorless
*Z* = 4	0.32 × 0.32 × 0.25 mm

### Data collection

**Table:**

Oxford Diffraction GEMINI S Ultra diffractometer	8649 independent reflections
Radiation source: Enhance (Mo) X-ray Source	7257 reflections with *I* > 2σ(*I*)
graphite	*R*_int_ = 0.031
Detector resolution: 16.0774 pixels mm^-1^	θ_max_ = 25.1°, θ_min_ = 3.0°
ω and φ scans	*h* = −16→15
Absorption correction: multi-scan (*CrysAlis RED*; Oxford Diffraction, 2007)	*k* = −19→12
*T*_min_ = 0.824, *T*_max_ = 0.859	*l* = −24→23
18204 measured reflections	

### Refinement

**Table:**

Refinement on *F*^2^	Secondary atom site location: difference Fourier map
Least-squares matrix: full	Hydrogen site location: inferred from neighbouring sites
*R*[*F*^2^ > 2σ(*F*^2^)] = 0.041	H-atom parameters constrained
*wR*(*F*^2^) = 0.108	*w* = 1/[σ^2^(*F*_o_^2^) + (0.0739*P*)^2^] where *P* = (*F*_o_^2^ + 2*F*_c_^2^)/3
*S* = 0.99	(Δ/σ)_max_ = 0.001
8649 reflections	Δρ_max_ = 0.60 e Å^−^^3^
580 parameters	Δρ_min_ = −0.32 e Å^−^^3^
0 restraints	Absolute structure: Flack (1983), 3798 Friedel pairs
Primary atom site location: structure-invariant direct methods	Flack parameter: −0.003 (12)

### Special details

**Table:**

Geometry. Bond distances, angles *etc*. have been calculated using the rounded fractional coordinates. All su's are estimated from the variances of the (full) variance-covariance matrix. The cell e.s.d.'s are taken into account in the estimation of distances, angles and torsion angles
Refinement. Refinement on *F*^2^ for ALL reflections except those flagged by the user for potential systematic errors. Weighted *R*-factors *wR* and all goodnesses of fit *S* are based on *F*^2^, conventional *R*-factors *R* are based on *F*, with *F* set to zero for negative *F*^2^. The observed criterion of *F*^2^ > σ(*F*^2^) is used only for calculating -*R*-factor-obs *etc*. and is not relevant to the choice of reflections for refinement. *R*-factors based on *F*^2^ are statistically about twice as large as those based on *F*, and *R*-factors based on ALL data will be even larger.

### Fractional atomic coordinates and isotropic or equivalent isotropic displacement parameters (Å^2^)

**Table:**

	*x*	*y*	*z*	*U*_iso_*/*U*_eq_	Occ. (<1)
Cu	0.81847 (3)	0.00134 (2)	0.36373 (2)	0.0308 (1)	
P1	0.69652 (6)	0.09021 (6)	0.37345 (4)	0.0349 (3)	
P2	0.71346 (6)	−0.10238 (6)	0.36133 (4)	0.0343 (3)	
P3	0.92358 (6)	0.04648 (5)	0.28923 (4)	0.0306 (3)	
P4	0.94056 (6)	−0.02611 (5)	0.43175 (4)	0.0313 (3)	
C111	0.6711 (3)	0.1520 (3)	0.30246 (16)	0.0448 (13)	
C112	0.6509 (3)	0.1130 (3)	0.2451 (2)	0.0700 (18)	
C113	0.6366 (4)	0.1602 (5)	0.1899 (2)	0.092 (3)	
C114	0.6426 (4)	0.2416 (5)	0.1922 (3)	0.091 (3)	
C115	0.6611 (4)	0.2795 (4)	0.2482 (3)	0.0820 (19)	
C116	0.6764 (3)	0.2352 (3)	0.30327 (17)	0.0550 (16)	
C121	0.6800 (3)	0.1617 (2)	0.43856 (15)	0.0399 (11)	
C122	0.7587 (4)	0.1953 (3)	0.4664 (2)	0.0647 (17)	
C123	0.7491 (4)	0.2558 (4)	0.5120 (3)	0.081 (2)	
C124	0.6629 (5)	0.2784 (3)	0.53131 (19)	0.079 (2)	
C125	0.5843 (4)	0.2465 (3)	0.5055 (2)	0.0690 (19)	
C126	0.5919 (3)	0.1870 (3)	0.45849 (19)	0.0520 (14)	
C131	0.5934 (3)	0.0255 (3)	0.3763 (2)	0.0504 (15)	
C211	0.7110 (3)	−0.1674 (3)	0.43170 (18)	0.0477 (14)	
C212	0.7035 (3)	−0.1306 (3)	0.4906 (2)	0.0687 (18)	
C213	0.7052 (4)	−0.1750 (6)	0.5461 (2)	0.110 (3)	
C214	0.7143 (4)	−0.2562 (6)	0.5434 (3)	0.103 (3)	
C215	0.7229 (4)	−0.2954 (4)	0.4854 (3)	0.093 (3)	
C216	0.7217 (3)	−0.2493 (3)	0.4287 (2)	0.0627 (18)	
C221	0.7001 (3)	−0.1710 (2)	0.29441 (15)	0.0368 (11)	
C222	0.7789 (3)	−0.1921 (2)	0.25936 (19)	0.0493 (12)	
C223	0.7721 (4)	−0.2460 (3)	0.2086 (2)	0.0660 (19)	
C224	0.6864 (4)	−0.2789 (3)	0.19210 (19)	0.0650 (18)	
C225	0.6083 (4)	−0.2588 (3)	0.2263 (2)	0.0620 (17)	
C226	0.6143 (3)	−0.2052 (3)	0.2770 (2)	0.0527 (14)	
C231	0.5998 (3)	−0.0527 (3)	0.3700 (2)	0.0493 (14)	
C311	0.9274 (3)	0.1571 (2)	0.28494 (16)	0.0360 (11)	
C312	0.9530 (4)	0.2000 (2)	0.33843 (19)	0.0580 (14)	
C313	0.9482 (4)	0.2831 (3)	0.3383 (2)	0.0773 (19)	
C314	0.9186 (4)	0.3241 (3)	0.2847 (2)	0.0647 (16)	
C315	0.8954 (3)	0.2820 (3)	0.2316 (2)	0.0553 (16)	
C316	0.8991 (3)	0.1984 (2)	0.23125 (17)	0.0430 (12)	
C321	0.9378 (2)	0.01646 (19)	0.20539 (14)	0.0334 (10)	
C322	0.8658 (3)	−0.0235 (2)	0.17519 (17)	0.0450 (12)	
C323	0.8760 (3)	−0.0481 (3)	0.11160 (18)	0.0547 (16)	
C324	0.9573 (3)	−0.0296 (2)	0.07897 (17)	0.0530 (14)	
C325	1.0295 (3)	0.0097 (3)	0.10925 (17)	0.0513 (14)	
C326	1.0200 (3)	0.0335 (2)	0.17224 (16)	0.0423 (11)	
C331	1.0380 (2)	0.0223 (2)	0.32486 (16)	0.0387 (11)	
C411	0.9581 (2)	−0.1319 (2)	0.45297 (17)	0.0365 (11)	
C412	0.9580 (3)	−0.1879 (3)	0.40355 (19)	0.0517 (14)	
C413	0.9655 (4)	−0.2689 (3)	0.4161 (3)	0.0680 (19)	
C414	0.9729 (3)	−0.2951 (3)	0.4785 (3)	0.0687 (19)	
C415	0.9750 (3)	−0.2406 (3)	0.5272 (2)	0.0633 (17)	
C416	0.9684 (3)	−0.1592 (3)	0.51499 (18)	0.0500 (14)	
C421	0.9642 (3)	0.0276 (2)	0.50622 (15)	0.0376 (11)	
C422	0.8886 (3)	0.0501 (3)	0.5431 (2)	0.0600 (14)	
C423	0.9031 (4)	0.0894 (4)	0.6010 (2)	0.075 (2)	
C424	0.9920 (4)	0.1067 (3)	0.62089 (19)	0.0660 (18)	
C425	1.0681 (4)	0.0827 (3)	0.5849 (2)	0.0670 (17)	
C426	1.0560 (3)	0.0430 (3)	0.52687 (18)	0.0530 (14)	
C431	1.0445 (2)	−0.0075 (3)	0.38339 (15)	0.0394 (10)	
O1	0.2511 (4)	−0.0101 (5)	0.2593 (3)	0.149 (3)	
C1	0.3146 (9)	0.0317 (7)	0.2194 (5)	0.166 (4)*	
C2	0.2957 (11)	0.0668 (9)	0.1717 (7)	0.106 (4)*	0.500
C3	0.3925 (19)	0.0228 (16)	0.2408 (11)	0.195 (9)*	0.500
F1	0.3819 (5)	−0.0475 (4)	0.4200 (5)	0.280 (5)	
F2	0.3857 (3)	0.0782 (3)	0.4354 (3)	0.177 (3)	
F3	0.2782 (4)	−0.0018 (4)	0.4846 (3)	0.180 (3)	
F4	0.2657 (4)	0.0418 (4)	0.3960 (3)	0.194 (3)	
B	0.3308 (3)	0.0080 (6)	0.4313 (4)	0.108 (3)	
H112	0.64680	0.05590	0.24330	0.0840*	
H113	0.62260	0.13440	0.15050	0.1100*	
H114	0.63370	0.27250	0.15440	0.1090*	
H115	0.66370	0.33680	0.24970	0.0980*	
H116	0.69050	0.26220	0.34210	0.0660*	
H122	0.81990	0.17710	0.45450	0.0780*	
H123	0.80370	0.28100	0.52930	0.0970*	
H124	0.65720	0.31780	0.56400	0.0950*	
H125	0.52380	0.26440	0.51920	0.0830*	
H126	0.53660	0.16400	0.44030	0.0620*	
H131	0.53290	0.04910	0.38260	0.0600*	
H212	0.69720	−0.07360	0.49290	0.0830*	
H213	0.70000	−0.14870	0.58620	0.1320*	
H214	0.71490	−0.28670	0.58180	0.1230*	
H215	0.72940	−0.35240	0.48370	0.1110*	
H216	0.72840	−0.27520	0.38840	0.0760*	
H222	0.83860	−0.16950	0.27000	0.0590*	
H223	0.82710	−0.26010	0.18510	0.0790*	
H224	0.68190	−0.31530	0.15720	0.0780*	
H225	0.54890	−0.28180	0.21540	0.0740*	
H226	0.55880	−0.19160	0.30020	0.0630*	
H231	0.54380	−0.08430	0.36980	0.0590*	
H312	0.97410	0.17230	0.37550	0.0700*	
H313	0.96530	0.31230	0.37560	0.0930*	
H314	0.91470	0.38140	0.28490	0.0780*	
H315	0.87630	0.31010	0.19430	0.0660*	
H316	0.88220	0.16950	0.19380	0.0520*	
H322	0.80880	−0.03460	0.19740	0.0540*	
H323	0.82690	−0.07730	0.09120	0.0660*	
H324	0.96340	−0.04420	0.03530	0.0640*	
H325	1.08640	0.02080	0.08690	0.0620*	
H326	1.07000	0.06160	0.19280	0.0510*	
H331	1.09400	0.03130	0.30090	0.0460*	
H412	0.95260	−0.16980	0.36070	0.0620*	
H413	0.96560	−0.30690	0.38210	0.0810*	
H414	0.97660	−0.35110	0.48750	0.0830*	
H415	0.98100	−0.25900	0.56990	0.0760*	
H416	0.97090	−0.12170	0.54920	0.0600*	
H422	0.82610	0.03880	0.52910	0.0720*	
H423	0.85050	0.10410	0.62660	0.0900*	
H424	1.00160	0.13540	0.65960	0.0790*	
H425	1.13020	0.09330	0.60000	0.0810*	
H426	1.10890	0.02690	0.50210	0.0630*	
H431	1.10510	−0.01940	0.40040	0.0470*	
H1	0.25560	0.00560	0.29910	0.1610*	
H2	0.25760	0.11240	0.18180	0.1270*	0.500
H3	0.35220	0.08420	0.15160	0.1270*	0.500
H4	0.26190	0.03200	0.14400	0.1270*	0.500
H5	0.39020	0.01980	0.28570	0.2330*	0.500
H6	0.42000	−0.02480	0.22380	0.2330*	0.500
H7	0.43090	0.06820	0.22860	0.2330*	0.500
H8	0.31070	0.01190	0.17710	0.1980*	0.500
H9	0.29950	0.08810	0.21940	0.1980*	0.500
H10	0.34180	0.07180	0.24620	0.1980*	0.500
H11	0.36040	−0.00660	0.20770	0.1980*	0.500

### Atomic displacement parameters (Å^2^)

**Table:**

	*U*^11^	*U*^22^	*U*^33^	*U*^12^	*U*^13^	*U*^23^
Cu	0.0238 (2)	0.0345 (2)	0.0341 (2)	0.0010 (2)	0.0012 (2)	−0.0039 (2)
P1	0.0287 (5)	0.0403 (5)	0.0356 (5)	0.0067 (4)	0.0004 (4)	−0.0073 (4)
P2	0.0292 (4)	0.0369 (5)	0.0368 (4)	−0.0038 (4)	0.0044 (4)	−0.0044 (4)
P3	0.0292 (4)	0.0324 (5)	0.0302 (4)	−0.0011 (4)	0.0001 (4)	−0.0004 (3)
P4	0.0258 (4)	0.0373 (5)	0.0309 (4)	0.0031 (4)	0.0008 (3)	−0.0004 (3)
C111	0.030 (2)	0.073 (3)	0.0313 (18)	0.016 (2)	0.0013 (16)	−0.0042 (17)
C112	0.052 (3)	0.109 (4)	0.049 (2)	0.015 (3)	−0.012 (2)	−0.019 (3)
C113	0.069 (4)	0.175 (7)	0.033 (2)	0.006 (4)	−0.006 (2)	−0.007 (3)
C114	0.063 (4)	0.152 (6)	0.059 (3)	0.030 (4)	0.010 (3)	0.035 (4)
C115	0.066 (3)	0.113 (4)	0.067 (3)	0.036 (3)	0.021 (3)	0.035 (3)
C116	0.059 (3)	0.067 (3)	0.039 (2)	0.013 (2)	0.0067 (19)	0.0075 (18)
C121	0.052 (2)	0.039 (2)	0.0286 (17)	0.0124 (19)	0.0032 (18)	−0.0014 (14)
C122	0.067 (3)	0.067 (3)	0.060 (3)	0.017 (3)	−0.011 (2)	−0.025 (2)
C123	0.087 (4)	0.078 (4)	0.078 (4)	0.014 (3)	−0.021 (3)	−0.034 (3)
C124	0.136 (6)	0.062 (3)	0.040 (2)	0.036 (4)	0.002 (3)	−0.016 (2)
C125	0.087 (4)	0.064 (3)	0.056 (3)	0.020 (3)	0.025 (3)	−0.010 (2)
C126	0.063 (3)	0.048 (2)	0.045 (2)	0.006 (2)	0.013 (2)	−0.0021 (18)
C131	0.0241 (18)	0.061 (3)	0.066 (3)	0.0063 (17)	0.0036 (17)	−0.016 (2)
C211	0.029 (2)	0.068 (3)	0.046 (2)	−0.0127 (19)	0.0020 (17)	0.013 (2)
C212	0.051 (3)	0.110 (4)	0.045 (2)	−0.028 (3)	0.006 (2)	0.004 (2)
C213	0.078 (4)	0.210 (9)	0.041 (3)	−0.064 (5)	0.003 (3)	0.028 (4)
C214	0.049 (3)	0.193 (8)	0.066 (4)	−0.029 (4)	−0.004 (3)	0.071 (5)
C215	0.045 (3)	0.115 (5)	0.119 (5)	0.005 (3)	0.007 (3)	0.072 (4)
C216	0.045 (2)	0.076 (4)	0.067 (3)	−0.001 (2)	0.009 (2)	0.024 (2)
C221	0.045 (2)	0.0310 (19)	0.0344 (17)	−0.0031 (16)	0.0002 (17)	0.0037 (14)
C222	0.054 (2)	0.046 (2)	0.048 (2)	−0.006 (2)	0.0079 (19)	−0.0105 (18)
C223	0.082 (4)	0.063 (3)	0.053 (3)	−0.003 (3)	0.025 (2)	−0.011 (2)
C224	0.103 (4)	0.047 (3)	0.045 (2)	−0.014 (3)	−0.003 (3)	−0.0107 (18)
C225	0.074 (3)	0.054 (3)	0.058 (3)	−0.021 (2)	−0.013 (2)	−0.008 (2)
C226	0.051 (2)	0.053 (3)	0.054 (2)	−0.011 (2)	−0.003 (2)	−0.007 (2)
C231	0.034 (2)	0.059 (3)	0.055 (2)	−0.0118 (18)	0.0073 (18)	−0.014 (2)
C311	0.0338 (19)	0.0351 (19)	0.0392 (19)	−0.0023 (16)	0.0033 (16)	−0.0013 (15)
C312	0.082 (3)	0.040 (2)	0.052 (2)	−0.017 (2)	−0.016 (2)	−0.0042 (18)
C313	0.098 (4)	0.054 (3)	0.080 (3)	−0.018 (3)	−0.014 (3)	−0.023 (3)
C314	0.080 (3)	0.036 (2)	0.078 (3)	−0.008 (2)	0.009 (3)	0.002 (2)
C315	0.068 (3)	0.037 (2)	0.061 (3)	0.000 (2)	0.003 (2)	0.004 (2)
C316	0.051 (2)	0.032 (2)	0.046 (2)	−0.0009 (17)	−0.0049 (17)	−0.0016 (16)
C321	0.0438 (19)	0.0275 (19)	0.0288 (16)	0.0029 (16)	0.0019 (15)	0.0008 (13)
C322	0.048 (2)	0.046 (2)	0.041 (2)	−0.0019 (18)	−0.0024 (17)	−0.0011 (16)
C323	0.072 (3)	0.051 (3)	0.041 (2)	−0.001 (2)	−0.014 (2)	−0.0109 (19)
C324	0.080 (3)	0.046 (2)	0.033 (2)	0.007 (2)	−0.001 (2)	−0.0043 (16)
C325	0.073 (3)	0.040 (2)	0.0410 (19)	0.003 (2)	0.0122 (19)	0.0074 (17)
C326	0.051 (2)	0.037 (2)	0.0389 (19)	−0.0021 (17)	0.0036 (16)	0.0025 (16)
C331	0.0279 (18)	0.049 (2)	0.0391 (19)	0.0044 (16)	0.0049 (14)	−0.0003 (16)
C411	0.0272 (18)	0.038 (2)	0.0442 (19)	0.0068 (15)	0.0027 (15)	0.0047 (15)
C412	0.048 (2)	0.052 (3)	0.055 (2)	0.012 (2)	−0.0040 (19)	−0.0080 (19)
C413	0.068 (3)	0.045 (3)	0.091 (4)	0.015 (2)	−0.011 (3)	−0.012 (2)
C414	0.049 (3)	0.044 (3)	0.113 (4)	0.008 (2)	0.022 (3)	0.013 (3)
C415	0.060 (3)	0.063 (3)	0.067 (3)	0.015 (2)	0.020 (2)	0.027 (2)
C416	0.056 (3)	0.048 (2)	0.046 (2)	0.014 (2)	0.0111 (19)	0.0098 (18)
C421	0.038 (2)	0.044 (2)	0.0309 (17)	0.0022 (16)	−0.0035 (14)	−0.0026 (14)
C422	0.045 (2)	0.085 (3)	0.050 (2)	0.012 (2)	−0.0030 (18)	−0.025 (2)
C423	0.063 (3)	0.110 (5)	0.053 (3)	0.015 (3)	−0.002 (2)	−0.027 (3)
C424	0.084 (4)	0.069 (3)	0.045 (2)	−0.003 (3)	−0.012 (2)	−0.019 (2)
C425	0.062 (3)	0.079 (3)	0.060 (3)	−0.017 (3)	−0.019 (2)	−0.010 (2)
C426	0.046 (2)	0.066 (3)	0.047 (2)	−0.005 (2)	−0.0038 (18)	−0.0040 (19)
C431	0.0237 (15)	0.052 (2)	0.0424 (18)	0.0074 (18)	0.0013 (13)	0.0037 (17)
O1	0.113 (4)	0.196 (6)	0.139 (4)	−0.048 (4)	0.033 (3)	−0.065 (4)
F1	0.168 (6)	0.134 (4)	0.537 (13)	0.075 (4)	0.217 (7)	0.132 (6)
F2	0.084 (3)	0.161 (5)	0.286 (7)	−0.022 (3)	0.072 (4)	−0.036 (5)
F3	0.145 (4)	0.180 (5)	0.215 (5)	0.021 (4)	0.096 (4)	0.071 (5)
F4	0.133 (4)	0.229 (7)	0.219 (6)	−0.014 (4)	−0.051 (4)	0.051 (5)
B	0.018 (2)	0.157 (7)	0.150 (6)	0.004 (4)	0.006 (3)	0.106 (6)

### Geometric parameters (Å, °)

**Table:**

Cu—P1	2.2775 (10)	C2—H9	1.0600
Cu—P2	2.2724 (11)	C2—H3	0.9500
Cu—P3	2.2820 (10)	C2—H2	0.9500
Cu—P4	2.2851 (10)	C3—H6	0.9500
P1—C111	1.841 (4)	C3—H5	0.9400
P1—C121	1.822 (3)	C3—H10	1.0900
P1—C131	1.812 (5)	C3—H11	0.9600
P2—C211	1.827 (4)	C3—H7	0.9600
P2—C221	1.815 (3)	C311—C312	1.376 (5)
P2—C231	1.816 (4)	C311—C316	1.376 (5)
P3—C311	1.836 (3)	C212—H212	0.9500
P3—C321	1.837 (3)	C312—C313	1.379 (6)
P3—C331	1.827 (3)	C313—C314	1.378 (6)
P4—C411	1.826 (3)	C213—H213	0.9500
P4—C421	1.827 (3)	C314—C315	1.354 (6)
P4—C431	1.812 (3)	C214—H214	0.9500
F1—B	1.194 (11)	C315—C316	1.386 (6)
F2—B	1.401 (10)	C215—H215	0.9500
F3—B	1.352 (10)	C216—H216	0.9500
F4—B	1.307 (9)	C321—C326	1.384 (5)
O1—C1	1.409 (13)	C321—C322	1.370 (5)
O1—H1	0.8800	C222—H222	0.9500
C111—C116	1.381 (7)	C322—C323	1.401 (5)
C111—C112	1.394 (6)	C323—C324	1.373 (6)
C112—C113	1.411 (7)	C223—H223	0.9500
C113—C114	1.353 (12)	C224—H224	0.9500
C114—C115	1.356 (9)	C324—C325	1.368 (6)
C115—C116	1.385 (8)	C325—C326	1.384 (5)
C121—C126	1.380 (6)	C225—H225	0.9500
C121—C122	1.375 (7)	C226—H226	0.9500
C122—C123	1.392 (8)	C331—C431	1.325 (5)
C123—C124	1.338 (9)	C231—H231	0.9500
C124—C125	1.345 (8)	C411—C416	1.384 (5)
C125—C126	1.398 (6)	C411—C412	1.391 (6)
C131—C231	1.306 (7)	C312—H312	0.9500
C1—C2	1.187 (18)	C412—C413	1.372 (7)
C1—C3	1.20 (3)	C313—H313	0.9500
C211—C216	1.367 (7)	C413—C414	1.382 (9)
C211—C212	1.381 (6)	C414—C415	1.363 (7)
C112—H112	0.9500	C314—H314	0.9500
C212—C213	1.376 (8)	C415—C416	1.376 (7)
C113—H113	0.9500	C315—H315	0.9500
C213—C214	1.353 (14)	C316—H316	0.9500
C214—C215	1.383 (10)	C421—C422	1.371 (6)
C114—H114	0.9500	C421—C426	1.393 (6)
C215—C216	1.413 (8)	C322—H322	0.9500
C115—H115	0.9500	C422—C423	1.392 (7)
C116—H116	0.9500	C423—C424	1.356 (8)
C221—C222	1.380 (6)	C323—H323	0.9500
C221—C226	1.388 (6)	C324—H324	0.9500
C122—H122	0.9500	C424—C425	1.373 (7)
C222—C223	1.392 (6)	C425—C426	1.393 (6)
C223—C224	1.374 (8)	C325—H325	0.9500
C123—H123	0.9500	C326—H326	0.9500
C224—C225	1.358 (7)	C331—H331	0.9500
C124—H124	0.9500	C412—H412	0.9500
C125—H125	0.9500	C413—H413	0.9500
C225—C226	1.387 (6)	C414—H414	0.9500
C126—H126	0.9500	C415—H415	0.9500
C131—H131	0.9500	C416—H416	0.9500
C1—H9	0.9600	C422—H422	0.9500
C1—H10	0.9500	C423—H423	0.9500
C1—H8	0.9500	C424—H424	0.9500
C1—H11	0.9400	C425—H425	0.9500
C2—H4	0.9500	C426—H426	0.9500
C2—H8	0.9400	C431—H431	0.9500
			
Cu···H112	3.6200	C326···H413^vii^	2.8900
Cu···H122	3.4800	C326···H331	2.8900
Cu···H212	3.4400	C426···C124^xii^	3.540 (7)
Cu···H222	3.4600	C426···C416	3.581 (7)
Cu···H312	3.6000	C331···H326	2.8800
Cu···H322	3.5400	C331···H312	2.8500
Cu···H412	3.4100	C431···F4^ix^	3.245 (6)
Cu···H422	3.5200	C421···H416	2.6300
P1···P2	3.2106 (15)	C422···H122	2.9700
P1···P3	3.7360 (13)	C422···H416	3.0800
P1···C231	2.736 (5)	C424···H6^vi^	2.8400
P2···P1	3.2106 (15)	C425···H8^vi^	3.0200
P2···P4	3.7546 (13)	C425···H11^vi^	3.0400
P2···C131	2.734 (5)	C425···H6^vi^	3.0700
P3···P1	3.7360 (13)	C426···H416	3.0200
P3···P4	3.2284 (13)	C426···H431	2.9300
P3···C431	2.760 (3)	C431···H412	3.0200
P4···P2	3.7546 (13)	C431···H426	2.7100
P4···P3	3.2284 (13)	B···H1	2.9700
P4···C331	2.750 (3)	H1···B	2.9700
F1···C231	3.257 (9)	H1···H331^i^	2.3300
F1···C131	3.355 (9)	H1···F4	2.1200
F2···C131	3.306 (6)	H1···H5	1.9400
F3···C426^i^	3.349 (7)	H2···H216^iii^	2.3800
F4···C431^i^	3.245 (6)	H3···H7	1.9800
F4···O1	2.998 (9)	H3···H6	2.5400
F1···H231	2.5900	H5···H1	1.9400
F1···H131	2.7800	H6···H424^viii^	2.5300
F2···H126	2.5700	H6···H3	2.5400
F2···H123^ii^	2.7100	H6···C425^viii^	3.0700
F2···H224^iii^	2.7900	H6···C424^viii^	2.8400
F2···H131	2.4100	H7···H3	1.9800
F3···H215^iv^	2.6000	H7···C225^iii^	3.0700
F3···H426^i^	2.4700	H8···C425^viii^	3.0200
F4···H1	2.1200	H8···H425^viii^	2.5200
F4···H224^iii^	2.7200	H9···C225^iii^	3.0700
F4···H431^i^	2.4900	H9···C224^iii^	2.8900
O1···F4	2.998 (9)	H10···C224^iii^	2.8200
O1···C331^i^	3.356 (7)	H10···C225^iii^	2.9500
O1···H331^i^	2.4800	H11···C425^viii^	3.0400
O1···H115^v^	2.8200	H112···C131	2.9300
C2···H216^iii^	2.9300	H112···Cu	3.6200
C115···C315	3.333 (7)	H113···H416^viii^	2.5100
C116···C126	3.556 (6)	H114···H415^viii^	2.4100
C116···C315	3.529 (6)	H115···O1^iii^	2.8200
C116···C316	3.546 (6)	H116···C126	3.0700
C122···C422	3.428 (7)	H116···C121	2.6200
C124···C426^ii^	3.540 (7)	H116···C122	2.9900
C125···C426^ii^	3.576 (7)	H122···Cu	3.4800
C125···C425^ii^	3.414 (7)	H122···C422	2.9700
C126···C116	3.556 (6)	H122···C312	3.1000
C131···F1	3.355 (9)	H123···F2^xii^	2.7100
C131···F2	3.306 (6)	H125···H312^ii^	2.5400
C211···C411	3.573 (5)	H126···H131	2.2600
C211···C412	3.560 (6)	H126···C131	2.7800
C212···C414^iv^	3.547 (6)	H126···F2	2.5700
C213···C414^iv^	3.363 (7)	H131···F1	2.7800
C114···H226^iii^	3.0600	H131···H126	2.2600
C214···C414^iv^	3.549 (7)	H131···F2	2.4100
C215···C414	3.540 (7)	H131···C126	2.9100
C215···C415^iv^	3.567 (7)	H212···Cu	3.4400
C216···C412	3.534 (6)	H212···C231	2.9400
C216···C413	3.474 (7)	H213···H316^vi^	2.5600
C216···C226	3.598 (6)	H215···F3^xi^	2.6000
C121···H116	2.6200	H216···H2^v^	2.3800
C122···H422	3.0600	H216···C221	2.6500
C222···C322	3.525 (5)	H216···C226	3.0700
C122···H116	2.9900	H216···C2^v^	2.9300
C126···H324^vi^	2.9700	H222···Cu	3.4600
C226···C216	3.598 (6)	H222···H412	2.4900
C126···H116	3.0700	H224···F4^v^	2.7200
C126···H131	2.9100	H224···F2^v^	2.7900
C131···H112	2.9300	H226···C231	2.7900
C231···F1	3.257 (9)	H226···H231	2.3100
C131···H126	2.7800	H226···C114^v^	3.0600
C315···C412^vii^	3.545 (6)	H231···F1	2.5900
C315···C116	3.529 (6)	H231···H226	2.3100
C315···C115	3.333 (7)	H231···C226	2.9600
C316···C116	3.546 (6)	H312···Cu	3.6000
C316···C326	3.453 (5)	H312···C331	2.8500
C221···H216	2.6500	H312···H125^xii^	2.5400
C322···C222	3.525 (5)	H313···C324^vii^	2.9900
C222···H322	2.9500	H314···C321^vii^	3.0700
C224···H10^v^	2.8200	H314···C326^vii^	2.8300
C224···H9^v^	2.8900	H316···H213^viii^	2.5600
C225···H424^viii^	2.9200	H316···C326	3.0100
C225···H10^v^	2.9500	H316···C321	2.6700
C225···H7^v^	3.0700	H322···Cu	3.5400
C225···H9^v^	3.0700	H322···C222	2.9500
C326···C316	3.453 (5)	H324···C126^viii^	2.9700
C226···H231	2.9600	H326···H331	2.3400
C226···H216	3.0700	H326···C331	2.8800
C331···O1^ix^	3.356 (7)	H331···O1^ix^	2.4800
C231···H226	2.7900	H331···C326	2.8900
C231···H212	2.9400	H331···H1^ix^	2.3300
C411···C211	3.573 (5)	H331···H326	2.3400
C312···H122	3.1000	H412···C315^x^	3.0000
C412···C211	3.560 (6)	H412···Cu	3.4100
C412···C315^x^	3.545 (6)	H412···C431	3.0200
C412···C216	3.534 (6)	H412···H222	2.4900
C413···C216	3.474 (7)	H413···C316^x^	3.0500
C414···C213^xi^	3.363 (7)	H413···C326^x^	2.8900
C414···C215	3.540 (7)	H413···C325^x^	3.0500
C414···C212^xi^	3.547 (6)	H414···C325^x^	3.0700
C414···C214^xi^	3.549 (7)	H415···H114^vi^	2.4100
C315···H412^vii^	3.0000	H416···C422	3.0800
C415···C215^xi^	3.567 (7)	H416···C426	3.0200
C416···C426	3.581 (7)	H416···C421	2.6300
C316···H413^vii^	3.0500	H416···H113^vi^	2.5100
C321···H316	2.6700	H422···C122	3.0600
C321···H314^x^	3.0700	H422···Cu	3.5200
C422···C122	3.428 (7)	H424···C225^vi^	2.9200
C324···H313^x^	2.9900	H424···H6^vi^	2.5300
C325···H413^vii^	3.0500	H425···H8^vi^	2.5200
C425···C125^xii^	3.414 (7)	H426···F3^ix^	2.4700
C325···H414^vii^	3.0700	H426···H431	2.2700
C426···C125^xii^	3.576 (7)	H426···C431	2.7100
C326···H314^x^	2.8300	H431···H426	2.2700
C326···H316	3.0100	H431···C426	2.9300
C426···F3^ix^	3.349 (7)	H431···F4^ix^	2.4900
			
P1—Cu—P2	89.76 (4)	H4—C2—H9	144.00
P1—Cu—P3	110.05 (3)	H5—C3—H7	109.00
P1—Cu—P4	130.20 (3)	H5—C3—H10	85.00
P2—Cu—P3	131.21 (3)	H5—C3—H11	133.00
P2—Cu—P4	110.94 (3)	C1—C3—H6	110.00
P3—Cu—P4	89.97 (3)	C1—C3—H5	110.00
Cu—P1—C111	115.80 (14)	H10—C3—H11	98.00
Cu—P1—C121	125.78 (13)	C1—C3—H10	49.00
Cu—P1—C131	103.31 (16)	C1—C3—H11	50.00
C111—P1—C121	102.61 (18)	C1—C3—H7	109.00
C111—P1—C131	101.5 (2)	H7—C3—H11	118.00
C121—P1—C131	104.89 (19)	H6—C3—H10	158.00
Cu—P2—C211	116.16 (14)	H5—C3—H6	110.00
Cu—P2—C221	123.99 (13)	H6—C3—H7	109.00
Cu—P2—C231	103.52 (16)	H6—C3—H11	60.00
C211—P2—C221	104.60 (18)	H7—C3—H10	79.00
C211—P2—C231	99.8 (2)	C312—C311—C316	119.1 (3)
C221—P2—C231	105.6 (2)	P3—C311—C312	118.9 (3)
Cu—P3—C311	112.33 (13)	P3—C311—C316	121.9 (3)
Cu—P3—C321	129.55 (10)	C213—C212—H212	119.00
Cu—P3—C331	103.06 (11)	C211—C212—H212	119.00
C311—P3—C321	102.73 (15)	C311—C312—C313	120.1 (4)
C311—P3—C331	102.29 (17)	C212—C213—H213	120.00
C321—P3—C331	103.56 (14)	C312—C313—C314	120.6 (4)
Cu—P4—C411	116.48 (11)	C214—C213—H213	120.00
Cu—P4—C421	125.03 (13)	C215—C214—H214	120.00
Cu—P4—C431	103.34 (11)	C313—C314—C315	119.3 (5)
C411—P4—C421	103.59 (16)	C213—C214—H214	120.00
C411—P4—C431	100.91 (18)	C214—C215—H215	121.00
C421—P4—C431	104.25 (18)	C314—C315—C316	120.7 (4)
C1—O1—H1	112.00	C216—C215—H215	120.00
P1—C111—C112	118.6 (4)	C215—C216—H216	120.00
C112—C111—C116	119.0 (4)	C211—C216—H216	120.00
P1—C111—C116	122.3 (3)	C311—C316—C315	120.2 (3)
C111—C112—C113	118.6 (5)	P3—C321—C326	121.1 (2)
C112—C113—C114	121.0 (5)	C322—C321—C326	119.5 (3)
C113—C114—C115	120.3 (6)	P3—C321—C322	119.4 (2)
C114—C115—C116	120.4 (6)	C221—C222—H222	120.00
C111—C116—C115	120.7 (4)	C223—C222—H222	120.00
P1—C121—C122	118.5 (3)	C321—C322—C323	120.2 (4)
P1—C121—C126	122.7 (3)	C224—C223—H223	120.00
C122—C121—C126	118.7 (4)	C222—C223—H223	120.00
C121—C122—C123	120.3 (5)	C322—C323—C324	119.7 (4)
C122—C123—C124	119.9 (5)	C223—C224—H224	120.00
C123—C124—C125	121.5 (5)	C323—C324—C325	120.1 (3)
C124—C125—C126	119.8 (5)	C225—C224—H224	120.00
C121—C126—C125	119.8 (4)	C224—C225—H225	120.00
P1—C131—C231	121.9 (3)	C324—C325—C326	120.4 (4)
C2—C1—C3	125.6 (17)	C226—C225—H225	120.00
O1—C1—C2	126.7 (13)	C321—C326—C325	120.1 (4)
O1—C1—C3	107.7 (14)	C225—C226—H226	120.00
P2—C211—C216	123.1 (3)	C221—C226—H226	119.00
C212—C211—C216	119.2 (4)	C131—C231—H231	119.00
P2—C211—C212	117.6 (4)	P3—C331—C431	121.4 (2)
C211—C212—C213	121.2 (5)	P2—C231—H231	119.00
C113—C112—H112	121.00	C412—C411—C416	118.8 (4)
C111—C112—H112	121.00	P4—C411—C416	123.9 (3)
C212—C213—C214	119.9 (5)	P4—C411—C412	117.4 (3)
C112—C113—H113	120.00	C311—C312—H312	120.00
C114—C113—H113	120.00	C313—C312—H312	120.00
C115—C114—H114	120.00	C411—C412—C413	120.7 (4)
C213—C214—C215	120.8 (6)	C314—C313—H313	120.00
C113—C114—H114	120.00	C312—C313—H313	120.00
C114—C115—H115	120.00	C412—C413—C414	119.7 (5)
C214—C215—C216	118.9 (6)	C413—C414—C415	120.1 (5)
C116—C115—H115	120.00	C313—C314—H314	120.00
C211—C216—C215	120.0 (4)	C315—C314—H314	120.00
C111—C116—H116	119.00	C316—C315—H315	120.00
C115—C116—H116	120.00	C414—C415—C416	120.6 (4)
P2—C221—C222	119.1 (3)	C314—C315—H315	120.00
P2—C221—C226	123.4 (3)	C315—C316—H316	120.00
C222—C221—C226	117.6 (3)	C411—C416—C415	120.2 (4)
C221—C222—C223	120.9 (4)	C311—C316—H316	120.00
C123—C122—H122	120.00	C422—C421—C426	120.2 (3)
C121—C122—H122	120.00	P4—C421—C426	121.7 (3)
C124—C123—H123	120.00	P4—C421—C422	118.1 (3)
C122—C123—H123	120.00	C321—C322—H322	120.00
C222—C223—C224	120.5 (5)	C421—C422—C423	120.2 (4)
C123—C124—H124	119.00	C323—C322—H322	120.00
C125—C124—H124	119.00	C324—C323—H323	120.00
C223—C224—C225	119.2 (4)	C422—C423—C424	120.2 (5)
C124—C125—H125	120.00	C322—C323—H323	120.00
C126—C125—H125	120.00	C323—C324—H324	120.00
C224—C225—C226	120.7 (5)	C423—C424—C425	119.8 (4)
C221—C226—C225	121.1 (4)	C325—C324—H324	120.00
C125—C126—H126	120.00	C326—C325—H325	120.00
C121—C126—H126	120.00	C324—C325—H325	120.00
C231—C131—H131	119.00	C424—C425—C426	121.3 (5)
P1—C131—H131	119.00	C321—C326—H326	120.00
P2—C231—C131	121.4 (3)	C421—C426—C425	118.2 (4)
O1—C1—H11	105.00	C325—C326—H326	120.00
H8—C1—H9	109.00	P4—C431—C331	121.6 (2)
C3—C1—H10	60.00	P3—C331—H331	119.00
C3—C1—H11	52.00	C431—C331—H331	119.00
O1—C1—H10	105.00	C411—C412—H412	120.00
O1—C1—H9	110.00	C413—C412—H412	120.00
O1—C1—H8	110.00	C414—C413—H413	120.00
H10—C1—H11	110.00	C412—C413—H413	120.00
C2—C1—H8	51.00	C413—C414—H414	120.00
C2—C1—H9	58.00	C415—C414—H414	120.00
C2—C1—H10	104.00	C416—C415—H415	120.00
C2—C1—H11	105.00	C414—C415—H415	120.00
C3—C1—H8	111.00	C415—C416—H416	120.00
C3—C1—H9	109.00	C411—C416—H416	120.00
H8—C1—H10	145.00	C423—C422—H422	120.00
H8—C1—H11	64.00	C421—C422—H422	120.00
H9—C1—H10	54.00	C422—C423—H423	120.00
H9—C1—H11	144.00	C424—C423—H423	120.00
H3—C2—H4	110.00	C423—C424—H424	120.00
H3—C2—H8	99.00	C425—C424—H424	120.00
C1—C2—H2	109.00	C426—C425—H425	119.00
H2—C2—H9	64.00	C424—C425—H425	119.00
H2—C2—H8	150.00	C421—C426—H426	121.00
C1—C2—H3	109.00	C425—C426—H426	121.00
C1—C2—H4	109.00	C331—C431—H431	119.00
C1—C2—H8	51.00	P4—C431—H431	119.00
C1—C2—H9	50.00	F1—B—F2	108.4 (5)
H2—C2—H3	110.00	F1—B—F3	113.9 (9)
H2—C2—H4	109.00	F1—B—F4	130.1 (9)
H8—C2—H9	101.00	F2—B—F3	110.7 (7)
H3—C2—H9	106.00	F2—B—F4	94.0 (7)
H4—C2—H8	66.00	F3—B—F4	97.5 (5)
			
P2—Cu—P1—C111	−107.91 (16)	C211—P2—C231—C131	119.5 (4)
P2—Cu—P1—C121	121.75 (15)	C221—P2—C231—C131	−132.2 (4)
P2—Cu—P1—C131	2.03 (14)	Cu—P3—C311—C312	60.4 (4)
P3—Cu—P1—C111	26.50 (17)	Cu—P3—C311—C316	−115.0 (3)
P3—Cu—P1—C121	−103.83 (15)	C321—P3—C311—C312	−156.6 (4)
P3—Cu—P1—C131	136.44 (14)	C321—P3—C311—C316	28.0 (4)
P4—Cu—P1—C111	134.42 (16)	C331—P3—C311—C312	−49.5 (4)
P4—Cu—P1—C121	4.08 (16)	C331—P3—C311—C316	135.2 (4)
P4—Cu—P1—C131	−115.65 (14)	Cu—P3—C321—C322	17.7 (3)
P1—Cu—P2—C211	−109.30 (16)	Cu—P3—C321—C326	−162.3 (2)
P1—Cu—P2—C221	118.66 (15)	C311—P3—C321—C322	−116.1 (3)
P1—Cu—P2—C231	−1.08 (14)	C311—P3—C321—C326	63.9 (3)
P3—Cu—P2—C211	133.82 (16)	C331—P3—C321—C322	137.7 (3)
P3—Cu—P2—C221	1.78 (16)	C331—P3—C321—C326	−42.3 (3)
P3—Cu—P2—C231	−117.96 (14)	Cu—P3—C331—C431	−5.3 (4)
P4—Cu—P2—C211	24.30 (17)	C311—P3—C331—C431	111.4 (3)
P4—Cu—P2—C221	−107.75 (15)	C321—P3—C331—C431	−142.1 (3)
P4—Cu—P2—C231	132.52 (14)	Cu—P4—C411—C412	47.5 (3)
P1—Cu—P3—C311	30.44 (14)	Cu—P4—C411—C416	−130.4 (3)
P1—Cu—P3—C321	−99.97 (13)	C421—P4—C411—C412	−171.2 (3)
P1—Cu—P3—C331	139.80 (11)	C421—P4—C411—C416	10.8 (3)
P2—Cu—P3—C311	138.73 (14)	C431—P4—C411—C412	−63.5 (3)
P2—Cu—P3—C321	8.32 (15)	C431—P4—C411—C416	118.6 (3)
P2—Cu—P3—C331	−111.91 (12)	Cu—P4—C421—C422	38.9 (4)
P4—Cu—P3—C311	−102.95 (14)	Cu—P4—C421—C426	−143.6 (3)
P4—Cu—P3—C321	126.65 (13)	C411—P4—C421—C422	−98.0 (3)
P4—Cu—P3—C331	6.41 (11)	C411—P4—C421—C426	79.6 (3)
P1—Cu—P4—C411	127.31 (13)	C431—P4—C421—C422	156.8 (3)
P1—Cu—P4—C421	−4.73 (16)	C431—P4—C421—C426	−25.7 (4)
P1—Cu—P4—C431	−123.09 (16)	Cu—P4—C431—C331	5.4 (4)
P2—Cu—P4—C411	18.79 (13)	C411—P4—C431—C331	126.2 (4)
P2—Cu—P4—C421	−113.25 (15)	C421—P4—C431—C331	−126.6 (4)
P2—Cu—P4—C431	128.39 (16)	P2—C211—C212—C213	−177.2 (4)
P3—Cu—P4—C411	−116.05 (12)	C216—C211—C212—C213	−1.0 (7)
P3—Cu—P4—C421	111.91 (15)	P2—C211—C216—C215	177.4 (4)
P3—Cu—P4—C431	−6.45 (16)	C212—C211—C216—C215	1.4 (7)
Cu—P1—C111—C112	57.1 (4)	C211—C212—C213—C214	0.0 (8)
Cu—P1—C111—C116	−119.4 (3)	C212—C213—C214—C215	0.6 (9)
C121—P1—C111—C112	−162.3 (3)	C213—C214—C215—C216	−0.2 (8)
C121—P1—C111—C116	21.3 (4)	C214—C215—C216—C211	−0.8 (7)
C131—P1—C111—C112	−54.0 (4)	P2—C221—C222—C223	178.5 (3)
C131—P1—C111—C116	129.6 (4)	C226—C221—C222—C223	−0.2 (6)
Cu—P1—C121—C122	33.8 (4)	P2—C221—C226—C225	−178.5 (3)
Cu—P1—C121—C126	−150.1 (3)	C222—C221—C226—C225	0.2 (6)
C111—P1—C121—C122	−101.5 (3)	C221—C222—C223—C224	0.5 (6)
C111—P1—C121—C126	74.6 (4)	C222—C223—C224—C225	−0.6 (7)
C131—P1—C121—C122	152.8 (3)	C223—C224—C225—C226	0.6 (7)
C131—P1—C121—C126	−31.1 (4)	C224—C225—C226—C221	−0.4 (7)
Cu—P1—C131—C231	−3.2 (4)	P4—C411—C412—C413	−176.4 (4)
C111—P1—C131—C231	117.1 (4)	C416—C411—C412—C413	1.7 (6)
C121—P1—C131—C231	−136.4 (4)	P4—C411—C416—C415	175.6 (3)
Cu—P2—C211—C212	50.4 (4)	C412—C411—C416—C415	−2.3 (6)
Cu—P2—C211—C216	−125.7 (3)	C411—C412—C413—C414	0.2 (7)
C221—P2—C211—C212	−169.1 (3)	C412—C413—C414—C415	−1.6 (7)
C221—P2—C211—C216	14.8 (4)	C413—C414—C415—C416	1.0 (7)
C231—P2—C211—C212	−60.0 (4)	C414—C415—C416—C411	1.0 (6)
C231—P2—C211—C216	123.9 (4)	P4—C421—C422—C423	178.4 (4)
Cu—P2—C221—C222	34.7 (3)	C426—C421—C422—C423	0.8 (7)
Cu—P2—C221—C226	−146.7 (3)	P4—C421—C426—C425	−178.6 (3)
C211—P2—C221—C222	−101.8 (3)	C422—C421—C426—C425	−1.1 (6)
C211—P2—C221—C226	76.9 (4)	C421—C422—C423—C424	0.9 (8)
C231—P2—C221—C222	153.4 (3)	C422—C423—C424—C425	−2.4 (8)
C231—P2—C221—C226	−27.9 (4)	C423—C424—C425—C426	2.2 (8)
Cu—P2—C231—C131	−0.6 (4)	C424—C425—C426—C421	−0.4 (7)

Symmetry codes: (i) *x*−1, *y*, *z*; (ii) *x*−1/2, −*y*+1/2, −*z*+1; (iii) −*x*+1, *y*+1/2, −*z*+1/2; (iv) *x*−1/2, −*y*−1/2, −*z*+1; (v) −*x*+1, *y*−1/2, −*z*+1/2; (vi) −*x*+3/2, −*y*, *z*+1/2; (vii) −*x*+2, *y*+1/2, −*z*+1/2; (viii) −*x*+3/2, −*y*, *z*−1/2; (ix) *x*+1, *y*, *z*; (x) −*x*+2, *y*−1/2, −*z*+1/2; (xi) *x*+1/2, −*y*−1/2, −*z*+1; (xii) *x*+1/2, −*y*+1/2, −*z*+1.

### Hydrogen-bond geometry (Å, °)

**Table:**

*D*—H···*A*	*D*—H	H···*A*	*D*···*A*	*D*—H···*A*
O1—H1···F4	0.8800	2.1200	2.998 (9)	179.00
C131—H131···F2	0.9500	2.4100	3.306 (6)	158.00
C331—H331···O1^ix^	0.9500	2.4800	3.356 (7)	153.00
C426—H426···F3^ix^	0.9500	2.4700	3.349 (7)	154.00
C431—H431···F4^ix^	0.9500	2.4900	3.245 (6)	137.00

Symmetry codes: (ix) *x*+1, *y*, *z*.
